# Management of chronic lung diseases in Sudan and Tanzania: how ready are the country health systems?

**DOI:** 10.1186/s12913-021-06759-9

**Published:** 2021-07-24

**Authors:** Uzochukwu Egere, Elizabeth Shayo, Nyanda Ntinginya, Rashid Osman, Bandar Noory, Stella Mpagama, ElHafiz Hussein, Rachel Tolhurst, Angela Obasi, Kevin Mortimer, Asma El Sony, Miriam Taegtmeyer, The IMPALA Consortium, Emmanuel Addo-Yobo, Brian Allwood, Hastings Banda, Imelda Bates, Amsalu Binegdie, Adegoke Falade, Jahangir Khan, Maia Lesosky, Bertrand Mbatchou, Hellen Meme, Beatrice Mutayoba, Louis Niessen, Jamie Rylance, William Worodria, Heather Zar, Eliya Zulu, Lindsay Zurba, S Bertel Squire

**Affiliations:** 1 Department of International Public Health, Liverpool School of Tropical Medicine, Liverpool, L3 5QA UK; 2 National Institute for Medical Research, Dar es Salaam, Tanzania; 3 NIMR-Mbeya Medical Research Center, Mbeya, Tanzania; 4 The Epidemiological Laboratory, Khartoum, Sudan; 5 Kibong’oto Infectious Diseases Hospital, Mae Street, Kilimanjaro, Tanzania; 6 Department of Clinical Sciences, Liverpool School of Tropical Medicine, Liverpool, UK; 7 Tropical Infectious Diseases Unit, Royal Liverpool University Hospital, Prescot Street, Liverpool, UK

## Abstract

**Background:**

Chronic lung diseases (CLDs), responsible for 4 million deaths globally every year, are increasingly important in low- and middle-income countries where most of the global mortality due to CLDs currently occurs. As existing health systems in resource-poor contexts, especially sub-Saharan Africa (SSA), are not generally oriented to provide quality care for chronic diseases, a first step in re-imagining them is to critically consider readiness for service delivery across all aspects of the existing system.

**Methods:**

We conducted a mixed-methods assessment of CLD service readiness in 18 purposively selected health facilities in two differing SSA health system contexts, Tanzania and Sudan. We used the World Health Organization’s (WHO) Service Availability and Readiness Assessment checklist, qualitative interviews of key health system stakeholders, health facility registers review and assessed clinicians’ capacity to manage CLD using patient vignettes. CLD service readiness was scored as a composite of availability of service-specific tracer items from the WHO service availability checklist in three domains: staff training and guidelines, diagnostics and equipment, and basic medicines. Qualitative data were analysed using the same domains.

**Results:**

One health facility in Tanzania and five in Sudan, attained a CLD readiness score of ≥ 50 % for CLD care. Scores ranged from 14.9 % in a dispensary to 53.3 % in a health center in Tanzania, and from 36.4 to 86.4 % in Sudan. The least available tracer items across both countries were trained human resources and guidelines, and peak flow meters. Only two facilities had COPD guidelines. Patient vignette analysis revealed significant gaps in clinicians’ capacity to manage CLD. Key informants identified low prioritization as key barrier to CLD care.

**Conclusions:**

Gaps in service availability and readiness for CLD care in Tanzania and Sudan threaten attainment of universal health coverage in these settings. Detailed assessments by health systems researchers in discussion with stakeholders at all levels of the health system can identify critical blockages to reimagining CLD service provision with people-centered, integrated approaches at its heart.

**Supplementary Information:**

The online version contains supplementary material available at 10.1186/s12913-021-06759-9.

## Introduction

Chronic lung diseases (CLDs) are diseases of the airways and structures of the lung including asthma, chronic obstructive pulmonary disease (COPD), bronchiectasis, occupational lung diseases and pulmonary hypertension [1]. They are responsible for a high burden of morbidity - especially from poorly controlled asthma - and mortality – especially from COPD – causing around 4 million deaths globally every year [2]. Post-tuberculosis lung disease (PTLD) is also increasingly recognised as an important cause of CLD, especially in high TB burden settings [3, 4]. An estimated 500 million people lived with CLD in 2017, an increase of 40 % compared to 1990 [5]. This has resulted in increased mortality - COPD is now the third most common cause of death globally [6]. COPD is expected to increase further as populations continue to age, including in low- and middle-income countries (LMICs) where 90 % of the global COPD mortality currently occurs [7] and the population is exposed to biomass use, smoking and occupational risk factors [8]. Though data are sparse, a systematic review of nine cross sectional studies from sub-Saharan Africa (SSA) showed prevalence of COPD ranging from 4.1 − 24.8 % [9]. Similarly, a review showed a wide variation in prevalence of asthma from 9.1 % in Ethiopia to 20.3 % in South Africa [10]. Interpretation of this variability is confounded by differences in study methodology and diagnostic approaches. This further affects recognition of the true burden of CLD.

CLDs therefore remain under recognised and poorly prioritised within the health systems in sub-Saharan Africa [11], with a paucity of data to guide decision making and policy by health systems stakeholders on management of CLDs in this setting. In Tanzania, data on CLD prevalence, diagnosis, management, and outcome are limited. A study of asthma among adolescents revealed prevalence of 17.6 and 6.4 % in urban and rural areas respectively [12]. Similarly, the prevalence of COPD in western rural Tanzania was found to be 18 % with a higher prevalence among men than women [13]. In Sudan, asthma incidence is rising among children in Khartoum state and the prevalence among adults was estimated to be around 10 % [14]. Unpublished CLD data has shown a COPD prevalence of 16.5 % in urban Sudan and a restrictive lung function was found in 55.6 % of adults who underwent spirometry. In addition, both Tanzania (high) and Sudan (moderate) have significant TB burdens which further increases the burden of CLDs as an association between TB and CLDs has been established [15, 16].

Existing systems are not generally oriented to provide quality care for CLD, especially with regard to chronic illness management [17]. A first step in re-imagining them is to critically consider readiness across all aspects of the existing system and how they interconnect [18]. It remains unclear how ready the health systems are to deliver quality CLD care in these settings. Several studies have examined the state of preparedness of health systems in LMICs to deliver general health services, specific disease services or primary health care in the context of universal health coverage [19–22]. Studies investigating preparedness for CLD services are however rare. A recent study found varied availability of spirometry and World Health Organization (WHO) essential medicines for COPD and asthma in Africa [23]. As part of a parent project exploring integration of CLD services into existing public health services, we assessed the readiness of health systems to deliver specialised CLD services in Tanzania and Sudan two different sub–Saharan African countries.

## Methods

This was a cross-sectional, mixed methods study that combined qualitative key informant interviews and healthcare worker in-depth interviews with a readiness assessment checklist, and review of TB registers and health facility records. Qualitative interviews were conducted to gain a detailed and nuanced description of the experiences of key health system players related to the characterization, prioritization and management of Chronic lung diseases. Clinical vignettes were included in the healthcare workers in-depth interviews to measure the knowledge of clinicians about CLD while at the same time measuring their skills in diagnosing and managing the condition.

### Health system contexts

The Sudan health system is administered at three levels: Federal, State and Locality [24]. Within Localities, Primary Health Services are provided at health centers in urban and rural settings, while within States, district hospitals provide secondary as well as referral services. National hospitals and State teaching hospitals provide tertiary care.

In Tanzania, the health system is administered as a pyramidal structure at five levels: the national, zonal, regional, district and ward/village levels. Primary health care services, the base of the pyramid, are provided at the ward/village level by health centers and dispensaries. Secondary health services are provided at the district level by the district hospitals while regional hospitals provide specialist referral services. Community-based health activities bring health promotion and prevention to families in villages or streets, often along with the support of vertical disease control programs, including the national TB program.

### Study sites

The study locations, Gezira State in Sudan and Dodoma region of Tanzania, were selected in collaboration with local Ministries of Health to allow “best-case” assessments. Gezira State was selected because of an existing pilot offering integrated services focusing on asthma standard case management. These services were developed by the Epidemiological laboratory (EPILAB), a research NGO with research infrastructure in Gezira collaborating in this study. Gezira state Ministry of Health has shown a strong interest in extending the existing pilot services. Dodoma Region was selected due to the strength of its TB Program and its strong community-based referral system for TB. In Tanzania, ten health facilities were purposively selected for readiness assessment to ensure inclusion of all health system levels, health facilities with and without TB diagnostic services, government-owned and non-government owned facilities, and both urban and rural settings. The participating sites were: one regional hospital, one district hospital, four health centers and four dispensaries. In Sudan eight district level facilities were selected: four where EPILAB asthma standard case management program is operational (‘EPILAB sites’) and 4 where this program is not yet operational (‘non EPILAB sites’); all eight were government owned as there were no privately owned health facilities in the study area at the same level as district hospitals. As a major objective of our study in Sudan is to scale up the existing asthma standard case management program to non EPILAB sites, only district level health facilities were selected. Furthermore, the district health facilities in Sudan receive direct referrals from the community and serve as the ‘close-to-community’ facilities equivalent to health centers in Tanzania.

### Readiness assessment and registers reviews

A structured assessment checklist based on the WHO Service Availability and Readiness Assessment (SARA) manual [25], was used to assess availability of human resources, treatment guidelines, drugs, equipment and community linkages in the facilities. All reported guidelines, equipment and medicines were visually checked. Readiness to deliver CLD services was defined as the ability of a health facility to provide out-patient care for chronic lung diseases, in accordance with the WHO SARA manual [25]. This was based on 3 domains of service availability and readiness:

- Staff training and guidelines: facility must have a guideline for CLD and the presence of at least one staff who received a refresher or in-service training on management of CLD in the past 2 years.
- Diagnostics and Equipment: facility must report availability of 4 equipment – peak flow meter, spacer devices, stethoscope and oxygen cylinder.
- Basic medicines: facility must report availability of 5 basic medicines - injectable epinephrine, hydrocortisone, prednisolone tablets, salbutamol and beclomethasone or other steroid inhaler.

Other equipment whose availability and functionality were explored included nebulizer with mask, pulse oximeter, sputum cups and glass slides.

Out-patient registers were reviewed in all facilities in both countries to identify CLD patients seen in the facilities in the previous month. In addition, records of the previous two years of the TB registers were reviewed in the TB clinics of 9 of the health facilities in Tanzania to determine the numbers diagnosed with TB and those without a TB diagnosis who could potentially have CLD. Any diagnosis of Asthma, Chronic Obstructive Pulmonary Disease (COPD), Chronic bronchitis, Bronchiectasis, Post TB lung disease or Occupational lung disease, was regarded as CLD. Diagnosis of these conditions was based on local practices including, clinical judgment of clinicians, limited investigations (where possible) and standard treatment guidelines.

### Participant selection for qualitative interviews

For the qualitative component, in-depth interviews (IDIs) were conducted with care providers, one from each of the selected health facilities in both countries. Healthcare workers were purposively identified from each health facility and included those who provided services for CLD patients such as medical doctors, non-medical doctor clinicians and nurses. In Tanzania, this included the TB focal persons and clinicians working in the specialised NCD clinic as CLD diagnosis is usually through TB clinic and the follow up conducted at the NCD clinic. In Sudan, selected healthcare workers included medical doctors and medical assistants.

Key informant interviews (KIIs) were conducted with purposively selected health system stakeholders from national, regional (or state in Sudan) and district levels and with in-depth knowledge of TB and CLD based on their job role.

### Data collection and analysis

All data were collected by authors (ES, RO, BN, HI) and research assistants trained in line with WHO SARA recommendations (WHO SARA ref, page 26 s. 1.5). Training covered overall aims of the study, data quality, facility data capture tools, how to administer questionnaire and record responses, ethical issues in health facility research, informed consent administration, privacy and confidentiality. Training included hands-on practical sessions and a data collection pilot in the health facility.

For the readiness assessment, research assistants visited the health facilities and interviewed the person in-charge of each facility using the structured readiness assessment checklist. For each service, the readiness score is computed as the mean availability of service-specific tracer items in the 3 domains: staff training and guidelines, diagnostics and equipment, and basic medicines. Equal weights are assigned to each of the 3 domains to achieve a total possible score of 100 % (i.e. 33.3 % each for staff training and guidelines, diagnostics and equipment, and basic medicines). Equal weights are also assigned to each domain indicator to contribute to the final total domain score of 33.3 % (16.7 % for each indicator in staff and training, 8.3 % for each indicator in diagnostics and equipment, and 6.7 % for each indicator in basic medicines).

Two levels of service readiness were defined:

- High readiness – health facility scored at least 50 % of the indicators in each of 3 domains.
- Low readiness – health facility scored less than 50 % of the indicators in each of 3 domains.

We presented service availability and readiness levels by facility and compared them across facilities and country where applicable. We performed simple descriptive analysis of the other quantitative data, with health facilities as units of analysis using frequencies.

Healthcare worker interviews used a structured topic guide to explore questions around knowledge and management of CLD and included a patient vignette to assess current skills and knowledge of CLD management and guidelines. The vignette simulated an outpatient consultation with a 60-year-old man with features of CLD, with specific questions posed to respondents about history, examination, diagnosis, treatment, and management of the patient. Respondents were requested to answer the questions and to fill an asthma diagnosis and treatment card at the end if their diagnosis was asthma. They were assured that this was not a test and that the result would not be shared with their supervisors or colleagues.

Similarly, key informant interviews were conducted using structured topic guides to explore overall readiness and topics including stakeholders’ prioritization of CLD services, service delivery, health workforce and the barriers to diagnosis and management of CLD patients.

Key informants and healthcare workers were interviewed at their offices and workspaces at a convenient time for them. Interviews were conducted in Arabic in Sudan, and in Swahili in Tanzania. All interviews were audio taped, transcribed verbatim and finally back translated into English by independent professional translators. The data was coded and analysed using a thematic framework approach [26].

### Ethical considerations

 The study was approved by the ethics committees of National Institute for Medical Research, Tanzania (ref /Vol.IX/2922); the Ministry of Health of Gezira state, Sudan (ref 44/T/KH/1) and the Liverpool School of Tropical Medicine (ref 18–043). Permission was also sought from the respective district authorities. Written informed consent was obtained from all participants in the qualitative interviews and from the in-charge of health facilities prior to the readiness assessment interview and completion of the checklist.

## Results

### Characteristics of health facilities

Altogether, 18 health facilities were visited for readiness assessment, 10 in Tanzania and 8 in Sudan.

The characteristics of the health facilities are shown in Table 1.

**Table 1:** Characteristics of health facilities assessed for readiness for chronic lung disease services in Tanzania and Sudan

	Tanzania (*n*=10)	Sudan (*n*=8)	Total
Type of facility			
Regional hospital	1	0	1
District hospital	1	8	9
Health centre	4	0	4
Dispensary	4	0	4
Managing authority			
Government/public	8	8	16
Mission/ faith-based	2	0	2
Private/NGO	0	0	0
Location			
Urban	1	1	2
Rural	9	7	16
Outpatient services only			
Yes	4	0	4

### General service readiness

#### Human resources

**Fig. 1:**
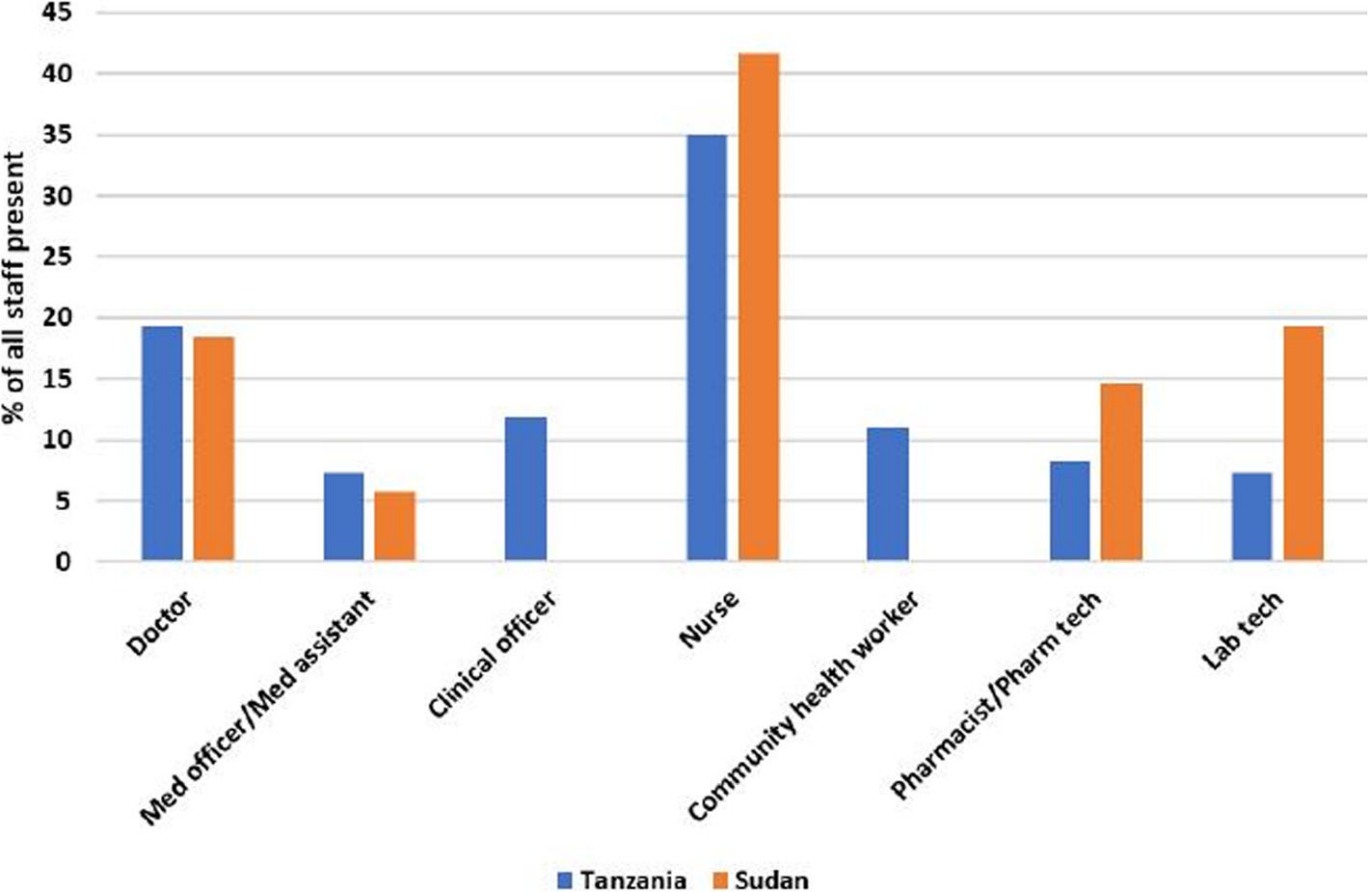
Human resource for generalised care as a percentage of all staff present on day of observation by cadre in Tanzania and Sudan

Nurses made up the majority of healthcare workers observed in the health facilities [38/109 (34.9 %) vs. 43/103 (41.7 %)] whilst assistant medical officers/medical assistants were the least available [8/109 (7.3 %) vs. 6/103 (5.8 %)] in Tanzania and Sudan respectively. Clinical officers and medical attendant cadres were unique to Tanzania (Fig. 1).

In Tanzania, clinical officers, nurses, and pharmacists/pharmacy technicians were the only healthcare staff cadres available in all facilities. All cadres were available at regional, district and health center levels. Of 21 medical doctors available on the day of facility visit, 16 (76.2 %) were at the regional hospital whilst only one (4.8 %) was at the health centers. Most of the nurses (15/38) were present at the district hospital. In Sudan, 19 medical doctors overall were present in all health facilities observed, with seven (36.8 %) of these in just one facility. Nurses and pharmacists/pharm tech were present in all health facilities.

#### Infrastructure and basic amenities

Outpatient-only services were offered at the four dispensaries in Tanzania. In the six facilities offering inpatient services, the bed capacity ranged from 22 in one of the health centers to 450 in the regional referral hospital. A functional ambulance for emergency transportation was available and operated from the facility in the regional hospital, the district hospital and in two of the health centers, but all health centers and dispensaries had access to a functional ambulance from another facility. A drug store was available in all facilities except two health centers and one dispensary. In Sudan, all facilities offered both inpatient and outpatient services and were equipped with drug stores with sufficient space. Bed capacity ranged from nine in the smallest facility to 128 in the largest. A functional ambulance was available in seven facilities, but three of the seven ambulance vehicles had no fuel on day of observation.

#### Service operation

A total of 19,812 OPD visits were recorded in all 10 health facilities in Tanzania over a period of one month, most of which occurred at the regional hospital. All 80 outpatient presumed TB cases were seen at two health centers and two dispensaries; 77 of these were found to be negative for TB. TB clinic registers reviewed in nine health facilities in Tanzania showed that 7,292 clients with presumed TB were investigated for TB over the previous two years; 955 (13.1 %) of these were diagnosed with TB whilst 6,355 (87.2 %) were TB negative. TB records were not available in one dispensary and therefore not reviewed in this facility. In contrast only two OPD visits were recorded for COPD over one-month period in all 10 facilities and this was at a health center.

In Sudan, 12,509 OPD visits overall were recorded in all 8 health facilities over the one-month review period. Asthma was responsible for 273 (2.2 %) of all OPD visits with 58 (21.2 %) of these in the four health facilities (‘EPILAB sites’) where EPILAB’s asthma management program is operational. There was no OPD visit identified as COPD over one-month period in any of the health facilities. Of the 38 presumed TB cases seen in six of the eight facilities, 13 were found to be negative for TB.

In both countries, asthma was far more likely to be diagnosed than COPD (119 vs. 2 in Tanzania, 392 vs. 0 in Sudan, respectively) during OPD visits. Of 32,321 total outpatient visits in one month across all 18 health facilities, only 2 was for COPD; this was in in a health center in Tanzania. The proportion of presumed TB among OPD visits is similar (0.4 % vs. 0.3 % in Tanzania and Sudan respectively), but the proportion of these patients shown to be TB negative was much higher in Tanzania (87.2 %) than in Sudan (34 %, 13/38). No final diagnosis on the TB negative patients could be traced and no diagnosis of post TB lung disease was found. A detailed analysis of the OPD visits is available as supplementary material (Additional file 1).

### CLD service readiness

#### Staff availability and training

No healthcare staff had received in-service or refresher training for CLD in any health facility in Tanzania in the past two years. However, four doctors, seven clinical officers and six nurses received pre-service training in CLD. In Sudan, staff who received in-service training in CLD were available in only 4 of the 8 health facilities. In three of these facilities, 1 doctor and 1 nurse were trained whilst just 1 doctor was trained in the remaining facility. Additionally, a medical assistant was trained in two of the facilities. No other cadre of staff received training in CLD.

#### Guidelines

In Tanzania, guidelines were more likely to be available at the health centers and dispensaries. None of the 10 guidelines identified as relevant in the study were available at either the regional or district hospitals. Only two facilities (one health center and one dispensary) had asthma guidelines. A COPD guideline was available in one health center. In Sudan, on the other hand, guidelines were more widely available. Asthma guidelines were available in five (62.5 %) of the eight health facilities, whilst pneumonia guidelines were available in four (50 %) facilities. In contrast, only 1 facility had a COPD guideline. A full list of the relevant guidelines and data on their availability in the health facilities are available as supplementary material (Additional file 2).

#### Equipment

In Tanzania, of all required essential equipment including peak flow meter, spacer devices, stethoscope and oxygen cylinder, only peak flow meters were unavailable in the regional and district hospitals. Stethoscopes were the only essential CLD equipment available in all facilities. In Sudan, all four items of equipment were available and functional in one facility, three in four facilities, and two in each of the remaining three facilities.

Nebulisers were available in two of the 10 facilities in Tanzania, and in seven of the eight facilities in Sudan. Sputum containers and microscope slides were available across all health facilities whilst oxygen sources were available in all health facilities except dispensaries in Tanzania.

#### Medicines

Salbutamol inhalers, epinephrine and hydrocortisone injections were widely available in both Tanzania and Sudan. On the other hand, steroid inhalers were not widely available. In Tanzania, beclomethasone was available only at the regional hospital, which also had fluticasone inhalers. In Sudan, where essential CLD medicines were more widely available, beclomethasone inhalers were available in only three of the facilities; in two of these three facilities, fluticasone and budesonide inhalers were additionally available. While salbutamol solution for nebulizer was available in all facilities in Sudan, it was only available in the district hospital in Tanzania. Prednisolone tablets were available in all health facilities.

#### Patient vignette

All clinicians who participated in the IDIs were offered the patient vignettes as part of the interviews. Fifteen healthcare workers, nine in Tanzania and six in Sudan, responded to the patient vignette during the IDIs. Two respondents in Tanzania were medical doctors, one at the regional hospital and one at the district hospital. One clinical officer declined. In Sudan, 2 medical doctors declined.

Overall, the respondents could correctly elicit an asthma history, describe the management of acute attacks, correct use of inhaler and patient counseling. Beyond these, only the medical doctors went further with the vignette. One medical doctor in Tanzania and five in Sudan made an initial diagnosis of asthma. All 6 identified the correct treatment for an acute asthmatic attack and antibiotic treatment for infections. Two medical doctors from Sudan made an initial diagnosis of COPD. However, when all doctors were presented with the next scenario where the patient returned 8 days later with persisting symptoms and new peak expiratory flow (PEF) values, only two understood PEF variability and only one of them could calculate it from the values provided. While most doctors considered excluding TB, COPD was not considered a differential diagnosis by any of those that made an initial diagnosis of asthma. Three doctors understood asthma severity grading but graded differently. Similarly, only 3 doctors mentioned psychological assessment for low mood in the simulated patient. When requested to fill an asthma management card, it was observed that asthma cards were not available in any of the health facilities in Tanzania. In Sudan, asthma cards were available only at EPILAB sites and the doctors were confident with completing them.

#### Readiness for CLD care at higher and lower health system levels

We conducted a separate analysis of the readiness scores in district level and above to compare CLD readiness across both countries at the higher health system level (Table 2). Overall, five health facilities (three EPILAB sites, two non EPILAB sites), all from Sudan, attained high readiness for providing CLD services. In Sudan, the lowest and highest scores were both at ‘EPILAB site’ facilities. No facility fulfilled all requirements for readiness in all the domains assessed. Staff with in-service or refresher training in CLD and beclomethasone inhaler were the two commonest missing tracer items overall; they were not available in 7 and 6 health facilities, respectively. In contrast, oxygen source was available in nine of the 10 facilities whilst stethoscope was available in all health facilities.

**Table 2:** CLD service readiness score (%) of regional and district level health facilities in Tanzania and Sudan

Readiness domain and indicators	Tanzania		Sudan							
	Regional	District	District1	District2	District3	District4	District5	District6	District7	District8
*Human resources and guidelines*										
i. CLD refresher or in-service training	0	0	16.7	16.7	16.7	0	0	0	0	0
ii. Guideline for any of COPD, asthma or chronic bronchitis	0	0	16.7	16.7	16.7	16.7	0	16.7	16.7	0
*Equipment*										
peak flow meter	0	0	0	0	8.3	0	0	8.3	8.3	0
spacer devices	0	0	0	8.3	8.3	8.3	0	0	0	8.3
stethoscope	8.3	8.3	8.3	8.3	8.3	8.3	8.3	8.3	8.3	8.3
oxygen cylinder	8.3	8.3	8.3	8.3	8.3	8.3	8.3	8.3	0	8.3
*Medicine*										
Inj. epinephrine	6.6	6.6	6.6	6.6	0	6.6	6.6	6.6	0	6.6
hydrocortisone injection	6.6	6.6	6.6	6.6	0	0	6.6	6.6	6.6	6.6
prednisolone tablets	6.6	6.6	6.6	6.6	6.6	0	0	6.6	6.6	6.6
salbutamol inhaler	6.6	6.6	0	6.6	6.6	6.6	6.6	0	0	0
beclomethasone inhaler	6.6	0	0	0	6.6	6.6	0	0	6.6	0
***Total readiness score***	**49.7**	**43.1**	**69.8**	**84.7**	**86.4**	**61.4**	**36.4**	**61.3**	**53.1**	**44.8**

Note: Total readiness score is the column total for all indicators in each of three domains (human resources and guidelines, equipment and medicine). Availability of an indicator in a health facility is scored 16.7, 8.3 and 6.6 in the three domains, respectively. Non availability is scored 0. All scores are in %.

Regarding CLD service readiness in health centers and dispensaries in Tanzania, one health center attained a total readiness score of 53.3 %. This health center had all CLD guidelines explored in the study. Of the remaining health facilities, four had scored 28.1 % each, two scored 21.1 % each and one scored 14.9 %. Overall, the average CLD service readiness score was 28.7 % in Tanzania and 62.3 % in Sudan.

#### Barriers to CLD care identified by key informants

Twelve key informants were interviewed in Tanzania whilst 13 were interviewed in Sudan. Key informants from both countries identified two major barriers to CLD care. First, CLDs were not regarded as priority diseases. In both countries, key informants regarded priority diseases as those with international recognition demonstrated by international funding and organized into programs such as HIV and TB: *“Such diseases attract high attention from the government since they are considered international programs, [so] have [the associated] weight, funds, international interest and evaluation, etc”* (KII 1, Federal, Sudan).

In Tanzania, none of the national level key informants mentioned CLDs among diseases considered as priority. NCDs, however, were mentioned as priority diseases but only with reference to Hypertension, Diabetes and Cancer: *“NCDs have masked these chronic lung diseases because we focus more on cancer, diabetes and cardiovascular conditions…. [As such] there is no priority especially when you meet NCD people, their focus is on diabetes and hypertensive diseases*” (KII, National, Tanzania).

Other diseases regarded as a priority included those commonly diagnosed in the outpatient departments and known locally to cause high mortality such as malaria and pneumonia in children, and maternal illnesses: *“Well, they have a high death rate, a high admission rate, and high frequency of patients, and they require a lot of effort from the Ministry of Health. … these are the standards we take into consideration, in addition to the financial toll they exert on the State and the government as a whole.”*. (K11 2, State, Sudan).

The second barrier identified by key informants was lack of data and this was key to low prioritization accorded CLD care by the authorities.

*“We can go to a policy-maker, and tell him or her that I need money because patients have increased…questions coming… to what extent have they increased? How much money do you need? Will that intervention really work? So, we must have data…if you have that then you can convince them, for example, for diabetes and hypertension we have taken those steps”* (KII 3, National, Tanzania). Health care workers, on the other hand, mentioned barriers already evident from the readiness assessment checklist. They mentioned lack of guidelines and lack of training as the major barriers to CLD care. The responses were similar in both countries.

## Discussion

In this study, we explored the readiness of health facilities to provide services for CLDs in two sub–Saharan African countries with differing health system contexts. We found that overall, no health facility fulfilled all the requirements for a CLD service with widespread gaps in human resources, guidelines, and equipment. However, six health facilities (1 in Tanzania and 5 in Sudan) attained high CLD service readiness. The high readiness in Sudan appeared to be driven by the EPILAB sites with existing asthma management programs. CLD was very rarely diagnosed, and our patient vignette analysis revealed significant gaps in capacity of clinicians to manage CLD. These findings were corroborated by qualitative data which also highlighted a lack of CLD data and low prioritization of CLD by health system actors, as barriers to CLD management.

Two previous studies in Tanzania have reported similar findings from a secondary data analysis of the 2014–2015 Tanzania Service Provision Assessment survey data. In one, only 10.9 % of over 700 facilities analysed, had high readiness for CLD services, and less than 10 % had at least one staff trained in management of CLDs [20]. In another, the mean CLD service readiness score of health facilities was 38.8 %, despite excluding facilities that did not report provision of CLD services [27]. Both studies found that CLD guidelines, peak flow meters and beclomethasone inhalers were the least available tracers for service readiness. These findings are similar in other SSA contexts. In Ethiopia, a NCD service availability and readiness assessment showed an average CLD readiness score of health facilities of 27 % [28], similar to the Tanzanian average (28.7 %) in our study. The additional use of patient vignettes in our study adds value by providing a snapshot of clinicians’ capacity to manage CLDs, not been reported by previous readiness studies. Additionally, poor prioritization of CLD by policymakers as shown in the key informant interviews reveals an important contributing factor for the poor readiness levels observed in this study.

Our study in Sudan focussed only on district level health facilities including those where an integrated approach to delivering a package of care for CLD is operational [29]. These facilities had peak flow meters and were more likely to have asthma guidelines, both of which were found to be frequently unavailable for CLD care in sub–Saharan African contexts [30]. However, experience with asthma care and overall higher readiness for CLD care did not translate to improved care of CLD in general, as seen in failure of the facilities to diagnose even a single case of COPD among over 12,000 adult patients seen over a month’s period. This highlights the need for a more comprehensive CLD training and availability of specific COPD guidelines.

In our study, trained staff, CLD guidelines, peak flow meters and beclomethasone inhalers were the most frequently missing tracer items, similar to findings from other studies [20, 27, 28, 31]. Lack of training is likely to have impacted the capacity of healthcare workers to recognise and diagnose CLD, especially COPD. Training of health professionals in respiratory disease has been identified as an important strategy in global control of chronic lung diseases [32], and remains a major gap in achieving and maintaining CLD service readiness in Tanzania and Sudan.

Lack of guidelines and trained staff observed in this study would lead to lack of capacity to recognise, diagnose and manage CLDs and therefore lack of data on which to base funding and planning for CLD care. As failure to provide funding for equipment and drugs would further lead to failure to make diagnosis,a potential vicious cycle could result which perpetuates negligence of CLD care within the health system and increased morbidity and mortality.

As health systems in LMICs face an increasing burden of NCDs, several studies have examined preparedness of health systems for chronic disease management. In most of these studies, service availability and readiness have been lowest in the context of CLDs. For example, in Uganda, health facilities were consistently less prepared for CLD services compared to other chronic diseases in most domains of service readiness [33]. Similarly, in a review of NCD service availability in first referral level hospitals in low-income countries, availability of medication and equipment for acute management was highest in epilepsy (40– 95 %) and hypertension (28 – 83 %) and lowest in asthma care (0–4 %) [34].This probably reflects the systemic negligence and poor investment in CLD by the health system as mentioned above. Of interest is the relatively high readiness for HIV care in most of the studies compared to NCDs. This probably reflects the impact of well-funded programs and could provide a model for strengthening CLD and other NCD services for policy makers in LMIC settings [18].

We found in Tanzania that health systems for CLD were weaker at the lower levels despite good potential to make a difference at this level. This has also been reported in previous studies within and outside Tanzania in the context of CLD [20, 27], hypertension and diabetes [19, 33]. This raises important equity concerns in Tanzania where about 70 % of the population lives in a rural setting [35] and are therefore served by the lower-level health facilities. By contrast, lower-level facilities are doing well in TB diagnosis. This creates the challenge of managing a large pool of chronic cough clients who have been found to be TB negative. In Malawi, only about 10 % of patients investigated in TB clinics were found to have TB disease [36]. In our study, the presumed TB patients seen in OPD were seen in the health centers and dispensaries. Of these, the 96 % who were TB negative, should ideally have been investigated to exclude CLD. There is therefore an urgent need for health policy makers to invest in staff training, diagnostics, and medicines to improve management of CLD patients in these countries.

### Limitations and strengths of the study

Our study did not use a standard survey strategy and the relatively limited numbers of health facilities we studied were purposively selected meaning our results may not be nationally representative. Nevertheless, our findings are consistent with studies in Tanzania and elsewhere which followed rigorous survey methodology [20, 27, 28]. However, it is possible that we may have over-estimated some aspects of readiness for CLD delivery. For example the SARA manual did not include a spirometer, an essential equipment in confirming the diagnosis of COPD [37]. Had this been included, none of the facilities in Tanzania would have achieved high readiness, whilst the high scoring facilities in Sudan would have been only marginally ready. It is also worthy of note that we have based the assessment of readiness of facilities on observations made on the day of study. Our use of vignettes to assess skill of clinicians may not adequately assess real clinical practice where, more nuances could impact clinicians’ responses and actions. Furthermore, the WHO readiness score may be too blunt an instrument to guide policy and planning as critical indicators that impact quality care such as staff training or guidelines could be lacking in the presence of ‘high facility readiness’. Our findings therefore need to be interpreted with caution.

However, our use of qualitative interviews allowed us to gain further insights from health systems actors. In addition, conducting the study across two African countries with differing health system contexts provided cross-country learning opportunities for both countries, and widened the scope of application of our study findings. Finally, the study was carried out primarily to inform development of interventions to integrate CLD care into existing health system. Therefore, the purposive selection of health facilities across health system levels where interventions are expected to be implemented, may be seen as a strength.

## Conclusions

Major gaps exist in service availability and readiness for CLD care in Tanzania and Sudan and threaten attainment of universal health coverage in these settings. Lack of guidelines, trained healthcare staff, peak flow meters and inhaled treatments (particularly bronchodilators and corticosteroids) for chronic airways diseases remain the most pressing need for facility readiness for CLD care across the health system. Detailed assessments by health systems researchers in discussion with stakeholders may be useful to identify critical blockages to re-orienting, or reimagining service provision with people-centered, integrated approaches at its heart.

## Supplementary Information

**Additional file 1.** Overall outpatient visits and visits for common CLDs, URTI and presumed TB over one-month by country and health facility.

**Additional file 2.** Availability of CLD guidelines, criteria for diagnosis and other guidelines by health facility level in Tanzania and Sudan

## Data Availability

Datasets used during this study are available from the corresponding author upon reasonable request.
