# Supplementary material for: Management of chronic lung diseases in Sudan and Tanzania: how ready are the country health systems?
Source: BMC Health Serv Res. 2021 Jul 24;21:734. doi: 10.1186/s12913-021-06759-9 (PMC8310588; doi:10.1186/s12913-021-06759-9)
Supplement: Supplementary file 1 — Additional file 1. Overall outpatient visits and visits for common CLDs, URTI and presumed TB over one-month by country and health facility. [file 12913_2021_6759_MOESM1_ESM.docx]

**Additional file 1:** Overall outpatient visits and visits for common CLDs, URTI and presumed TB over one-month by country and health facility

| Country and health facility | OPD visit for any conditions in previous month | OPD visit for specific conditions and their percentage contributions to all OPD visits in the health facility in one month, n (%) | | | | |
| --- | --- | --- | --- | --- | --- | --- |
|  | Total | Asthma | COPD | URTI | Presumed TB | TB neg |
| ***Tanzania***  Regional  District  Health centre1  Health centre2  Health centre3  Health centre4  Dispensary1  Dispensary2  Dispensary3  Dispensary4  **Total** | 15058  1819  766  202  258  317  675  83  353  281  **19812** | 70 (0.5)  27 (1.5)  4 (0.5)  3 (1.5)  3 (1.2)  3 (0.9)  0  0  0  0  **119 (0.6)** | 0  0  0  0  0  2 (0.6)  0  0  0  0  **2** | 71 (0.5)  0  134 (17.5)  49 (24.3)  0  76 (23.9)  1 (0.2)  6 (7.2)  48 (13.6)  147 (52.3)  **532 (2.7)** | 0  0  0  0  27 (10.5)  12 (3.8)  4 (0.6)  0  37 (10.5)  0  **80 (0.4)** | 0  0  0  0  24 (9.3)  12 (3.8)  4 (0.6)  0  37 (10.5)  0  **77 (0.4)** |
| ***Sudan***  District1  District2  District3  District4  District5  District6  District7  District8  **Total** | 995  2321  942  1633  2596  1386  1578  1058  **12509** | 0  50 (2.2)  19 (2.0)  30 (1.8)  6 (0.2)  18 (1.3)  15 (0.9)  135 (12.8)  **273 (2.2)** | 0  0  0  0  0  0  0  0  **0** | 35 (3.5)  138 (5.9)  84 (8.9)  68 (4.2)  65 (2.5)  124 (8.9)  362 (22.9)  70 (6.62)  **946 (7.6)** | 8 (0.8)  2 (0.1)  0  5 (0.3)  0  8 (0.6)  9 (0.6)  6 (0.57)  **38 (0.3)** | 2 (0.2)  0  0  3 (0.2)  0  2 (0.1)  3 (0.2)  3 (0.28)  **13 (0.1)** |
