# Supplementary material for: Management of chronic lung diseases in Sudan and Tanzania: how ready are the country health systems?
Source: BMC Health Serv Res. 2021 Jul 24;21:734. doi: 10.1186/s12913-021-06759-9 (PMC8310588; doi:10.1186/s12913-021-06759-9)
Supplement: Supplementary file 2 — Additional file 2. Availability of CLD guidelines, criteria for diagnosis and other guidelines by health facility level in Tanzania and Sudan [file 12913_2021_6759_MOESM2_ESM.docx]

**Additional file 2:** Availability of CLD guidelines, criteria for diagnosis and other guidelines by health facility level in Tanzania and Sudan

|  | Tanzania | | | | Sudan |
| --- | --- | --- | --- | --- | --- |
| Guideline | Regional (n=1) | District (n=1) | H. centre (n=4) | Dispensary (n=4) | District (n=8) |
| Pneumonia guideline | 0 | 0 | 1 | 2 | 4 |
| Asthma guideline | 0 | 0 | 1 | 1 | 5 |
| COPD guideline | 0 | 0 | 1 | 0 | 1 |
| Chronic bronchitis guideline | 0 | 0 | 1 | 0 | 2 |
| Criteria for referral | 0 | 0 | 1 | 1 | 4 |
| Criteria for diagnosis pneumonia | 0 | 0 | 1 | 2 | 4 |
| Criteria for diagnosis asthma | 0 | 0 | 1 | 2 | 4 |
| Criteria for COPD | 0 | 0 | 1 | 1 | 1 |
| Follow up COPD | 0 | 0 | 1 | 0 | 0 |
| Follow up asthma | 0 | 0 | 1 | 0 | 4 |

Numbers in cells represent number of facilities in which each guideline is available
